# Supplementary material for: Iodine-Modified Ag NPs for Highly Sensitive SERS Detection of Deltamethrin Residues on Surfaces
Source: Molecules. 2023 Feb 10;28(4):1700. doi: 10.3390/molecules28041700 (PMC9967755; doi:10.3390/molecules28041700)
Supplement: Supplementary file 1 [file molecules-28-01700-s001.zip › molecules-2129941-supplementary.pdf]

Iodine-Modified Ag NPs for Highly Sensitive SERS Detection of Deltamethrin Residues on Surfaces

Zhangmei Hu [1], Dandan Peng [2], Feiyue Xing [2], Xiang Wen [2], Kun Xie [3], Xuemei Xu [4], Hui Zhang [5], Feifei Wei [1], Xiaoke Zheng [1] and Meikun Fan [2,*]

[1] The Analysis and Testing Center, Southwest Jiaotong University, Chengdu 610031, China
[2] Faculty of Geosciences and Environmental Engineering, Southwest Jiaotong University
[3] Physical Science and Technology, Southwest Jiaotong University, Chengdu 610031, China
[4] Sichuan Academy of Environmental Sciences, Chengdu 610041, China
[5] Sichuan Zhongbiao Technology Co., Ltd., Chengdu 610052, China
* Correspondence: mkfan@swjtu.edu.cn

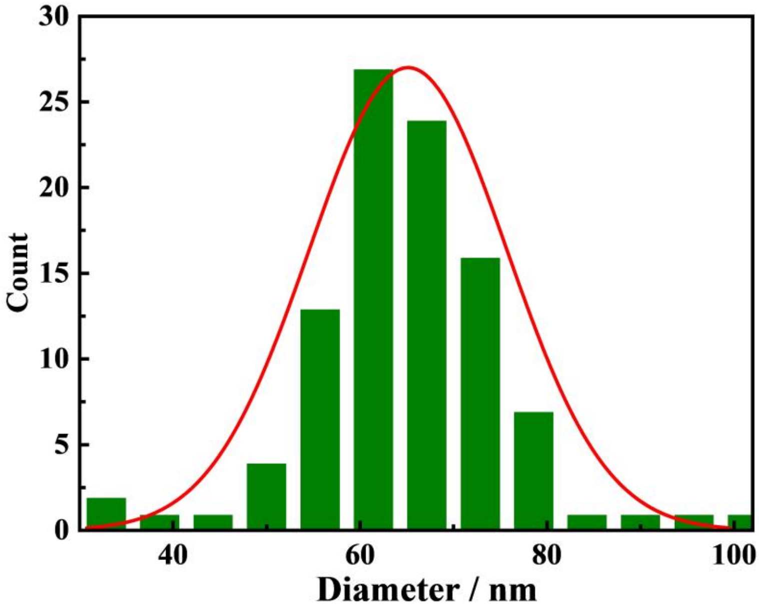

Figure S1. Nano measure of Ag NPs.

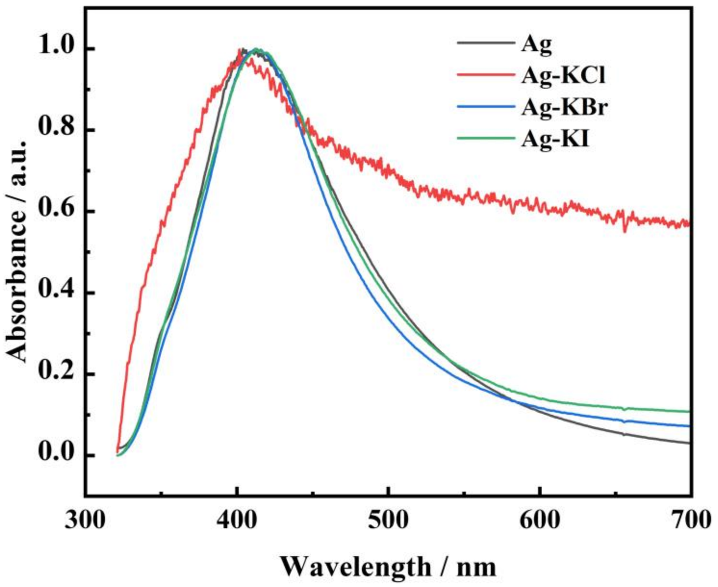

Figure S2. The UV spectra of Ag and the mixture of Ag NPs and 0.1 M KCl (red line), KBr (blue line) and KI (green line).

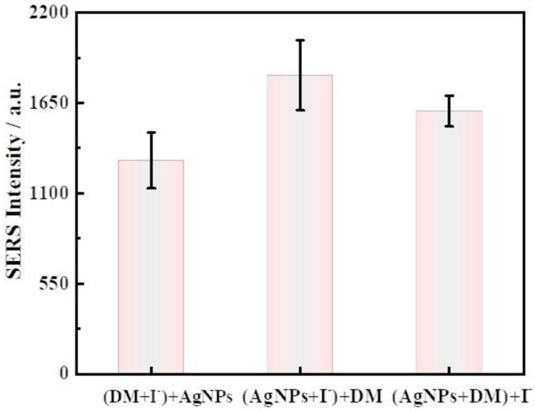

Figure S3. The SERS performance of different mixing orders of Ag NPs, KI and DM.

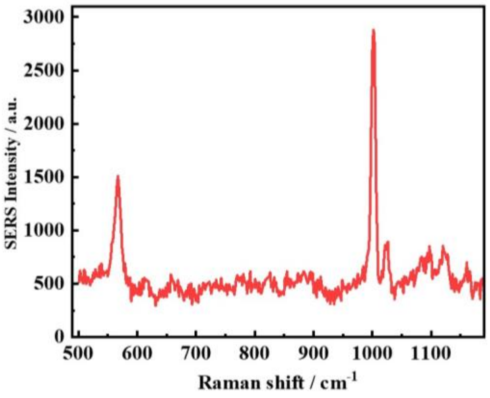

Figure S4. The SERS performance of commercial DM product diluted with methanol.

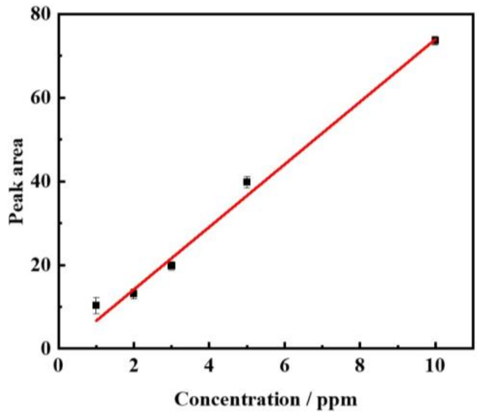

Figure S5. Standard curve of deltamethrin for HPLC. The concentrations of DM were 1 ppm, 2 ppm, 3 ppm, 5 ppm, 10 ppm, respectively. The fitting equation was y = 0.332 + 7.360 x (R^2 = 0.990).

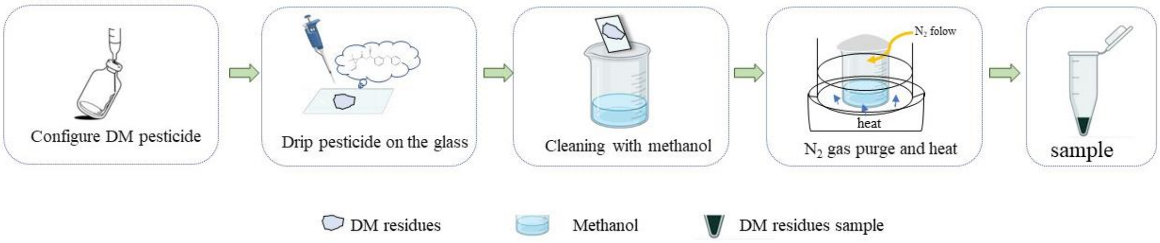

Figure S6. The diagram of setup for surface residue analysis.

Table S1. The assignment of SERS peaks of DM with Iodide assisted Ag NPs FSM sensor.

SERS (cm^{-1})	Powder(cm^{-1})	Assignment
563	563	υ(C-Br)ip +
1000	999	υring+δ(C-C)ip
1165	1165	δ(C-H)ip
1207	1207	υ(C-C)ip
1735	1740	υ(C=O)ip
2253	2254	υ(C=N)ip

Table S2. Statistical results of the SERS signal of eight Ag NPs FSMs.

FSMs	Mean intensity	RSD (%)
1	1196.5	11.6
2	1183.5	11.3
3	1277.5	11.7
4	1360.5	12.7
5	1110	7.1
6	1199.5	10.9
7	1144.3	6.7
8	1170.5	4.5

Table. S3 Analysis of the variance of results of SERS and HPLC method (n = 3).

Sample	Deltamethrin insecticide (ug/mL)	SS	df	MS	F	P-value	F crit
A	1	49.91	3	15.9	3.97	0.16	8.95
B	2.5	65.04	3	20.8	3.82	0.13	8.95
C	5	89.23	3	31.2	1.29	0.41	8.95
